# Supplementary material for: Path sampling of recurrent neural networks by incorporating known physics
Source: Nat Commun. 2022 Nov 24;13:7231. doi: 10.1038/s41467-022-34780-x (PMC9700810; doi:10.1038/s41467-022-34780-x)
Supplement: Supplementary file 1 — Supplementary Information [file 41467_2022_34780_MOESM1_ESM.pdf]

Supplementary Information for "Path sampling of recurrent neural networks by incorporating known physics"

Sun-Ting Tsai,[1] Eric Fields,[2] Yijia Xu,[3,4,5] En-Jui Kuo,[3,4] and Pratyush Tiwary*[6,a)]

[1)] *Department of Physics and Institute for Physical Science and Technology, University of Maryland, College Park 20742, USA.*

[2)] *Department of Chemistry and Biochemistry and Department of Computer Science, University of Maryland, College Park 20742, USA.*

[3)] *Department of Physics, University of Maryland, College Park 20742, USA.*

[4)] *Joint Quantum Institute and Joint Center for Quantum Information and Computer Science, NIST/University of Maryland, College Park, Maryland 20742, USA*

[5)] *Institute for Physical Science and Technology, University of Maryland, College Park 20742, USA.*

[6)] *Department of Chemistry and Biochemistry and Institute for Physical Science and Technology, University of Maryland, College Park 20742, USA.*

(Dated: 7 November 2022)

[a)] Electronic mail: ptiwary@umd.edu

Supplementary Note I: Markov dynamics details

The time series used for training with the 3 state Markov dynamics was generated through random sampling using the transition probability matrix shown below in Eq.1. The length of the time series used for input was 300000 units. The transition probability matrix P used to generate 3-state Markov dynamics is given by:

$$P = \begin{bmatrix} 0.9300 & 0.0667 & 0.0033 \\ 0.0667 & 0.8667 & 0.0667 \\ 0.0033 & 0.0667 & 0.9300 \end{bmatrix} \tag{1}$$

Predictions with the ps-LSTM when trained with the 3-state Markov Dynamics yielded the following transition matrix when constraining the first nearest neighbor transition rate

$$P = \begin{bmatrix} 0.8886 \pm 0.00039 & 0.1078 \pm 0.00039 & 0.0036 \pm 0.00004 \\ 0.0833 \pm 0.00057 & 0.8380 \pm 0.00105 & 0.0787 \pm 0.00056 \\ 0.0084 \pm 0.00009 & 0.1029 \pm 0.00047 & 0.8887 \pm 0.00050 \end{bmatrix} \tag{2}$$

where the estimate of $\langle N \rangle$ in Eq. 8 for the subset we selected is 0.1348. In Supplementary Fig. 1, we have shown the plot showing comparison of each transition probability calculated from input trajectory, prediction from unconstrained LSTM, analytical results calculated from Eq. 14, and prediction from ps-LSTM.

Supplementary Note II: Molecular dynamics and neural network details

The MD trajectory for Aib9 was obtained using the software GROMACS 5.0.4[1,2], patched with PLUMED 2.4[3]. The Aib9 molecule consists of 129 atoms solvated with 1540 TIP3P[4,5] water molecules. CHARMM36m30 all atom force field is used to parametrize the Aib9. Molecular dynamics (MD) was performed to generate the time-series, with temperature 500K using the Nose-Hoover thermostat[6] and the pressure maintained at ambient pressure with Parrinello-Rahman barostat[7]. The molecular dynamics integration time step is $2fs$.

For training an LSTM to learn the 3-state Markov dynamics, we took the embedding dimension $M = 8$ and the LSTM unit $L = 128$. The time series were batched into sequences with a sequence length of 35 and the batch size of 64. The models were trained with the method of stochastic gradient descent for 10 epochs. After sampling 100 LSTM predictions

a

$p_{transition}$	Input	LSTM	Theory	ps-LSTM	Error
p_{00}	0.9300	0.9339±0.0002	0.8853	0.8886±0.0004	0.3%
p_{01}	0.0670	0.0635±0.0002	0.1118	0.1078±0.0004	-3.6%
p_{02}	0.0030	0.0027±0.0000	0.0029	0.0036±0.0001	24.1%
p_{10}	0.0658	0.0671±0.0001	0.1098	0.0833±0.0006	-24.1%
p_{11}	0.8672	0.8664±0.0003	0.8255	0.8380±0.0011	-1.5%
p_{12}	0.0670	0.0665±0.0002	0.1118	0.0787±0.0006	-29.6%
p_{20}	0.0032	0.0037±0.0003	0.0030	0.0084±0.0001	180%
p_{21}	0.0677	0.0768±0.0004	0.1130	0.1029±0.0005	-8.9%
p_{22}	0.9291	0.920±0.0004	0.8845	0.8887±0.0005	0.5%

b

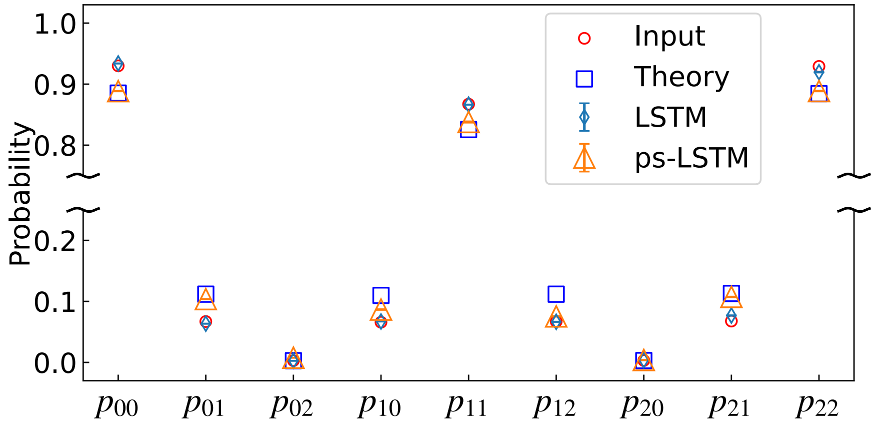

Supplementary Figure 1 3-state Markovian system: LSTM, ps-LSTM and analytical predictions Here we show results of applying ps-LSTM to the 3 state Markovian system where we constrain $\langle N \rangle$. In (a), we show the comparison of the transition probabilities from state-m to n, p_{mn}, between the input trajectory used to train our network, the predicted values given from analytical results in Appendix (Eq. 19), and the actual transition probability obtained via direct counting using the 200 predictions by ps-LSTM. The errors represent "error percentages" which are calculated as $(p_{\rm Theory} - p_{\rm ps-LSTM})/p_{\rm Theory}$. In (b), we plot the corresponding transition probabilities in (a).

of length 100 with $\Delta\lambda$ = -56.1 defined in Sec II 3 in main text, a ps-LSTM was retrained with

the same hyperparameters except for increasing epochs to 350. For training LSTM to learn Aib9, we discretized the input into 32 states, took the embedding dimension $M = 32$ and the LSTM unit $L = 64$. The time series were batched into sequences with a sequence length of 100 and the batch size of 64. The model was trained with the method of stochastic gradient descent for 40 epochs. The subsets consist of 10 selected trajectories for constraining the Aib9 MD simulations. The ps-LSTM was then retrained with exactly the same hyperparameters.

For training LSTM to learn the quantum jump trajectories, we took the embedding dimension $M = 16$ and the LSTM unit $L = 64$. The time series were batched into sequences with a sequence length of 100 and batch size of 64. The models were trained with the method of stochastic gradient descent for 20 epochs. After sampling 20,000 LSTM predictions of length 500, a ps-LSTM was retrained with the same hyperparameters except for increasing sequence length to 140 and 60 epochs.

Supplementary Note III: LSTM recurrence structure

As mentioned in the main text, the LSTM recurrence structure takes $\mathbf{x}^{(t)}$ and $\mathbf{h}^{(t-1)}$ as input to predict the next time step hidden state $\mathbf{h}^{(t)}$. At each time step, the layer is defined with the following equations of forward propagation:

$$\mathbf{f}^{(t)} = \sigma(\mathbf{W}_f\mathbf{x}^{(t)} + \mathbf{U}_f\mathbf{h}^{(t-1)} + \mathbf{b}_f) \tag{3}$$

$$\mathbf{i}^{(t)} = \sigma(\mathbf{W}_i\mathbf{x}^{(t)} + \mathbf{U}_i\mathbf{h}^{(t-1)} + \mathbf{b}_i) \tag{4}$$

$$\mathbf{o}^{(t)} = \sigma(\mathbf{W}_o\mathbf{x}^{(t)} + \mathbf{U}_o\mathbf{h}^{(t-1)} + \mathbf{b}_o) \tag{5}$$

$$\tilde{\mathbf{c}}^{(t)} = \tanh(\mathbf{W}_c\mathbf{x}^{(t)} + \mathbf{U}_c\mathbf{h}^{(t-1)} + \mathbf{b}_c) \tag{6}$$

$$\mathbf{c}^{(t)} = \mathbf{f}^{(t)} \circ \mathbf{c}^{(t-1)} + \mathbf{i}^{(t)} \circ \tilde{\mathbf{c}}^{(t)} \tag{7}$$

$$\mathbf{h}^{(t)} = \mathbf{o}^{(t)} \circ \tanh(\mathbf{c}^{(t)}) \tag{8}$$

where the $\mathbf{f}^{(t)}$, $\mathbf{i}^{(t)}$, $\mathbf{o}^{(t)}$, $\tilde{\mathbf{c}}^{(t)}$, and $\mathbf{c}^{(t)}$ are forget gate, input gate, output gate, candidate value, and the cell state, respectively. $\mathbf{W}$ and $\mathbf{U}$ are matrices defined for each gate.

Supplementary Note IV: Eigenspectrum of transition matrix

In this section, we have shown the eigenspectrum of the transition probability matrices obtained by counting from the trajectories of reference $200ns$ MD simulation of Aib9, the

LSTM prediction, and the ps-LSTM prediction.

In Supplementary Fig. 2, we can see that both ps-LSTM and LSTM capture the first four slow modes. While there are some deviations at 4th and 5th modes, the deviations mainly come from the training of LSTM. The ps-LSTM method performs path sampling from the predicted trajectories of LSTM therefore it simply captures the errors from the LSTM itself.

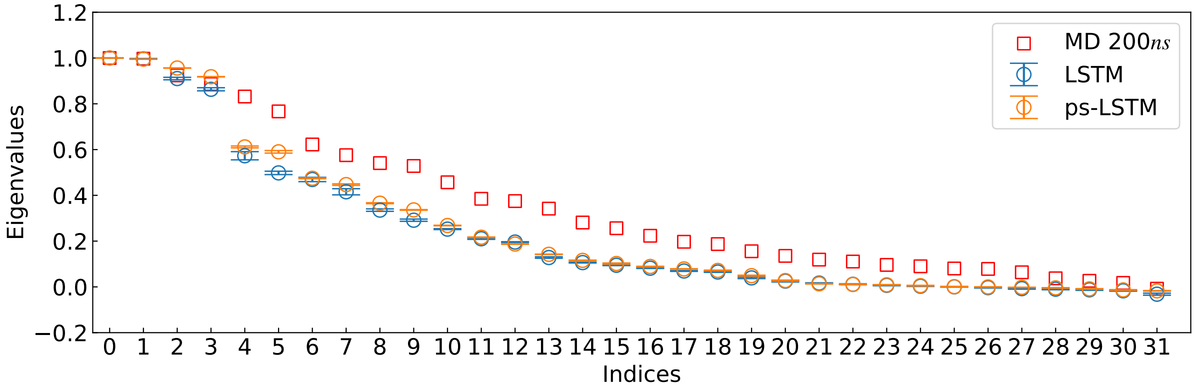

Supplementary Figure 2 Eigenspectrum of transition probability matrices In this plot, we show the eigenspectrum of the transition probability matrices calculated via direct counting from the reference $200ns$ MD simulation of Aib9 (red squares), the LSTM prediction (blue circles), and the ps-LSTM prediction (orange circles). The errorbars are calculated as standard errors.

Supplementary Note V: Transition time

The averaged transition times of Aib9 was calculated using a commitment time analysis. In the analysis, we first split the $200ns$ trajectory or $200ns$ predictions by LSTM or ps-LSTM into 10 or 20 segments, respectively. Various commitment time windows are then selected to detect whether there is a transition within these windows for each segment. The commitment time window serves as the timescale for excluding short timescale fluctuations. The detection is performed by checking whether a transition within the commitment time window undergoes a back-and-forth transition. If a transition can be identified as a back-and-forth transition, it is not committed or counted as a valid transition for the size of commitment time window chosen. As we increase the commitment time window size, the number of transitions would strictly decrease with various plateau. Since the L and R helix

located at $\chi \approx 5.4$ and $\chi \approx -5.4$, their corresponding state labels are i=10 and i=21, where χ_i is the discretized order parameter. As a result, the number of transitions corresponds to the plateau happened after 10 time steps and are larger than 3 time step is used to determine the number of transitions between L and R helix states.

The mean transition time was calculated as the total length of trajectory divided by the total number of transitions summed over each segments. The standard deviation was then calculated over the mean transition times of all segments.

Supplementary Note VI: Determining the size of subset size for subsampling

In this section, we describe our criterion for determining the size of subset for path sampling. In Supplementary Fig. 3, we have shown a test of 200 independent sampling trials with subset consisting of 10 predicted trajectories sampled using our method described in Sec. II 3 and constraints defined in Sec. III. For κ, we perform the sampling with the subset drawn from 200 predicted trajectories generated from constraint-free LSTM, while for $\langle N \rangle$ the total number of predicted trajectories produced by constraint-free LSTM is 800. As can be seen, the fluctuations of the averaged values calculated from the subsets are small enough to be distinguished from the actual values calculated from input training dataset. Therefore, we conclude that subset of size 10 is sufficient. The actual values of κ and $\langle N \rangle$ of the subsets we used are κ=1.010, 0.380 for constraint $\langle N \rangle$=0.38 and 0.42 for constraint $\langle N \rangle$=0.42.

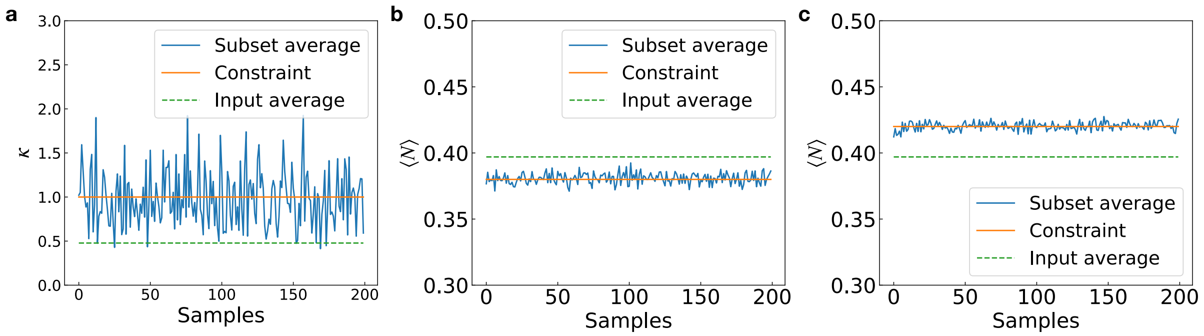

Supplementary Figure 3 Subset size for subsampling In this plot, we show the test independently sampling 200 subsets of size 10. (a) shows results for the averaged κ with constraint $\kappa = 1$, (b) for average $\langle N \rangle$ with constraint $\langle N \rangle = 0.38$, and (c) for average $\langle N \rangle$ with constraint $\langle N \rangle = 0.42$. In each plot, we also show the constraint values and the values calculated from input training dataset as orange solid and green dashed horizontal lines.

Supplementary References

[1] Herman JC Berendsen, David van der Spoel, and Rudi van Drunen. Gromacs: a message-passing parallel molecular dynamics implementation. Comp. Phys. Commun., 91(1-3): 43–56, 1995.

[2] Mark James Abraham, Teemu Murtola, Roland Schulz, Szilárd Páll, Jeremy C Smith, Berk Hess, and Erik Lindahl. Gromacs: High performance molecular simulations through multi-level parallelism from laptops to supercomputers. SoftwareX, 1:19–25, 2015.

[3] Massimiliano Bonomi, Giovanni Bussi, and Carlo Camilloni Camilloni. Promoting transparency and reproducibility in enhanced molecular simulations. Nat. Methods., 16:670–673, 2019.

[4] Alex D MacKerell Jr, Donald Bashford, MLDR Bellott, Roland Leslie Dunbrack Jr, Jeffrey D Evanseck, Martin J Field, Stefan Fischer, Jiali Gao, H Guo, Sookhee Ha, et al. All-atom empirical potential for molecular modeling and dynamics studies of proteins. The journal of physical chemistry B, 102(18):3586–3616, 1998.

[5] William L Jorgensen, Jayaraman Chandrasekhar, Jeffry D Madura, Roger W Impey, and Michael L Klein. Comparison of simple potential functions for simulating liquid water. The Journal of chemical physics, 79(2):926–935, 1983.

[6] Denis J Evans and Brad Lee Holian. The nose–hoover thermostat. The Journal of chemical physics, 83(8):4069–4074, 1985.

[7] Michele Parrinello and Aneesur Rahman. Polymorphic transitions in single crystals: A new molecular dynamics method. Journal of Applied physics, 52(12):7182–7190, 1981.
